# Gratifying Gizmos for Research and Clinical MEG

**DOI:** 10.3389/fneur.2021.814573

**Published:** 2022-01-27

**Authors:** Veikko Jousmäki

**Affiliations:** ^1^Aalto NeuroImaging, Department of Neuroscience and Biomedical Engineering, Aalto University, Espoo, Finland; ^2^Cognitive Neuroimaging Centre, Lee Kong Chian School of Medicine, Nanyang Technological University, Singapore, Singapore

**Keywords:** accelerometer, audiotactile, illusion, somatosensory, motor, stimulation

## Abstract

Experimental designs are of utmost importance in neuroimaging. Experimental repertoire needs to be designed with the understanding of physiology, clinical feasibility, and constraints posed by a particular neuroimaging method. Innovations in introducing natural, ecologically-relevant stimuli, with successful collaboration across disciplines, correct timing, and a bit of luck may cultivate novel experiments, new discoveries, and open pathways to new clinical practices. Here I introduce some gizmos that I have initiated in magnetoencephalography (MEG) and applied with my collaborators in my home laboratory and in several other laboratories. These gizmos have been applied to address neuronal correlates of audiotactile interactions, tactile sense, active and passive movements, speech processing, and intermittent photic stimulation (IPS) in humans. This review also includes additional notes on the ideas behind the gizmos, their evolution, and results obtained.

## Introduction

Although life is multisensory in nature, it is worth investigating sensory modalities with dedicated stimulators separately with neuroimaging methods. For this purpose, we need natural, ecologically-relevant stimuli which stimulate each sensory modality specifically and do not interfere with the neuroimaging modality used.

For example, magnetoencephalography (MEG) and electroencephalography (EEG) provide us tools to evaluate a given sensory system and its neuronal correlates and use the results in clinical assessments as guided in the clinical practice guidelines (1). For example, electric stimulation applied over the peripheral nerve is preferred to elicit somatosensory-evoked potentials (SEPs) and somatosensory-evoked fields (SEFs). Such a strong and non-specific sensory stimulus works perfectly to address neuroscientific questions, for example, what is the given peak latency and where the corresponding cortical representation is located. To be honest, such an approach works in most of the cases perfectly. However, such a sensory stimulus does not stimulate peripheral mechanoreceptors, for example, Pacinian corpuscles, proprioceptors, and slowly conducting tactile fibers specifically. Thus, we may miss some specific attributes in stimulation to address more detailed possibilities in basic research and clinical practice.

Why do not we have ecologically relevant, naturalistic stimulators in use in MEG? The answer is very simple—they are not commercially available. Availability may be limited due to the estimated market size which is typically considered to be not large enough for introducing new stimulators taking into account regulatory processes needed for medical devices.

There are two main approaches to introduce a novel stimulator in MEG. One approach is to use an original idea in basic research, apply it with an in-house built and locally-approved stimulator within a small number of subjects as investigator-initiated studies, and publish the results. The other approach is to file invention disclosures and proceed with patent applications to protect the immaterial property rights (IPRs) first and then proceed to collect the evidence in a multicenter study with the documented stimulator. Both approaches will take time and effort without any promise of the final outcome.

Here, I will present a few cases that I have initiated in MEG and, together with my collaborators, used successfully to discover novel findings. I have used mostly the basic research approach in introducing gizmos for MEG research.

## MEG Market

Before entering to the gizmos, it is worth checking the status quo in MEG including the market size, market forecast, main vendors, and stimulators. With more than 200 MEG devices in active use, non-invasive MEG plays a vital role in basic research and clinical applications. The clinical use of MEG is presented in recent surveys (2–7). With two clinical applications, namely presurgical functional mapping and localizing of epileptic foci, MEG is very useful in epilepsy and presurgical evaluation. Although MEG performed only in a fraction of epilepsy patients, it has a huge potential in epilepsy centers.

At present, all commercially available whole-head MEG systems approved by US Food and Drug Administration (FDA) are using superconducting quantum interference (SQUID) technology with liquid helium. Whole-head MEG systems utilizing optically pumped magnetometer (OPM) technology are now available for research purposes (8–13).

The main market areas for MEG are Northern America, Europe, and Asia. MEG market is gradually expanding. According to a recent market review by Verified Market Research (https://www.verifiedmarketresearch.com/product/magnetoencephalography-market), the MEG market size was valued ~200 million USD in 2020 and is projected to reach ~300 million USD by 2028. The main fuels for the market rise are the prevalence of brain diseases and growing popularity due to its non-invasive nature. The MEG market growth is estimated to be driven by the increase of MEG centers and advancements in OPM technology.

Main MEG vendors, for example, CTF (CTF MEG Neuro Innovations Inc, Coquitlam, BC, Canada; http://ctf.com), MEGIN (MEGIN Oy, Helsinki, Finland; http://megin.fi), NeuroScan (Compumedics Limited, Abbotsford, Victoria, Australia; https://compumedicsneuroscan.com), and Ricoh (Ricoh USA Inc., Tustin, CA; https://www.ricoh-usa.com), typically list a limited number of validated stimulators. These stimulators for visual, somatosensory, and auditory modalities have been tested according to the regulatory requirements concerning medical devices. Here, it is the FDA since the main market resides in the USA. Local approvals, for example, CE marking in the European Economic Area, may also be required. FDA-approved stimulators are typically provided by another vendor selling these devices also for other functional neuroimaging modalities, and these stimulators have been tested as a part of the MEG system.

Typically, a stimulation system in MEG is controlled with a commercial software package, for example, Presentation (Neurobehavioral Systems Inc., Berkeley, CA; https://www.neurobs.com), Stim2 (Compumedics Limited, Abbotsford, Victoria, Australia; https://compumedicsneuroscan.com), or E-Prime (Psychology Software Tools, Inc., Pittsburgh, PA; https://pstnet.com/). Many experienced and technically strong MEG teams have their own in-house built or third-party stimulators and software in use, for example, PsychoPy (https://psychopy.org) and Psychophysics Toolbox (http://psychtoolbox.org). Given the efforts needed for FDA clearance, third-party software packages and toolboxes are typically more flexible for research-oriented MEG compared with FDA-cleared software packages.

## Designing Gizmos

Magnetoencephalography and electroencephalography share the same origin of signals and temporal resolution. These aspects at theoretical, instrumentational, mathematical, and practical levels are depicted in details in Hari and Puce (14) and Hämäläinen et al. (15). Most of the commercially available stimulators and monitoring devices used commonly in other neuroimaging modalities, for example, functional magnetic resonance imaging (fMRI) and EEG, are not readily MEG compatible. Why MEG is so vulnerable to interferences? MEG sensors are very prone to magnetic and radiofrequency fields—this is the main reason for using the magnetically shielded room (MSR) in MEG to suppress ambient electromagnetic noise and to keep MEG sensors within their dynamic range.

Artifacts in MEG include several sources inside and outside the MSR, for example, ambient noise, various physiological signals, movement artifacts, and intrinsic MEG noise (16). Here, we focus on those elicited by stimulators and monitoring devices. Interfering artifacts inside the MSR may be elicited by magnetic materials moving close to MEG sensors, electric currents, ground loops, and radiofrequency disturbances associated with a given stimulator. For example, magnetic materials close to the MEG sensors combined with deep breathing, task-related movements, utterances, and ballistocardiographic body movements may give rise to disturbing artifacts in MEG. Here, the distance really matters—devices next to the MEG sensors need to be carefully tested for possible magnetic artifacts whereas devices fixed on the floor at a distance from the MEG sensors may contain some magnetic particles. Although noise suppression methods, for example, high-pass filtering, may help to attenuate, these low-frequency artifacts in MEG signals of interest may overlap with, for example, movement frequency. In such cases, more advanced noise suppression algorithms, for example, the temporal extension of signal space separation (17), maybe useful to attenuate artifacts leaving brain signals intact (18, 19).

It is important to note that implanted stimulators, for example, cardiac pacemakers, deep brain stimulators, and vagal nerve stimulators, contain magnetic particles and will cause severe artifacts in MEG. Given the dynamic range of the modern superconducting MEG systems, MEG measurements are feasible although off-line processing is needed to separate artifacts from the brain signals (20–23).

Digital signals cause RF interferences, and thus, analog signals are preferred inside the MSR. Cables entering the MSR should be filtered to rule out any potential RF interference since cables may act as antennas bringing external RF interference to the MSR. Direct current battery-operated devices are preferred to reduce possible interferences and ground loops to avoid deteriorated MEG signal quality.

Safety aspects and regulations concerning medical devices need to be taken into account, too.

Sensor manufactures, vendors, and suppliers provide huge selection of sensors and materials to choose from finding suitable ones for MEG purposes which take testing, time, effort, and luck. Vendors and suppliers do not specify MEG compatibility, and non-magnetic does not necessarily mean non-magnetic in MEG. Materials should be tested and selected carefully, and an MEG device can be utilized to find suitable materials since it picks up magnetic disturbances easily.

Material selection and manufacturing processes are of major importance in MEG. Most materials, for example, wood, plastic, and metals, can be used in MEG which provided that they are non-magnetic. However, some dyes are magnetic, and some materials typically considered to be non-magnetic, for example, aluminum and copper, may turn out to be magnetic due to the recycling processes introducing magnetic deposits. Manufacturing processes may also introduce magnetic artifacts, for example, a chrome-tipped solder iron will leave magnetic chrome deposits in soldering whereas copper tip does not cause such problems. In addition, some manufacturing processes, for example, modern gold-plating technique with magnetic nickel sublayer, cause major disturbances in MEG. Once again, the distance matters. A gold-plated EEG electrode typically introduces artifacts in MEG since it will be next to the MEG sensors and it will move with respect to the MEG sensors due to breathing, task-related movements, utterances, and ballistocardiographic movements whereas a gold-plated connector fixed on the floor of the MSR can easily be used without any artifacts in MEG.

Taking all these together, a novel stimulator or monitoring device has to be safe and easy to use, fulfill the local regulations, have local approvals, compatible with existing MEG systems, and should synchronize with the MEG data acquisition and stimulation systems precisely. As a physicist, I would like to say that the task is well-defined and feasible. Let me now introduce some gizmos.

## Audiotactile Interactions

Investigational approaches and their evolutions in multisensory interaction studies are well-covered in multisensory textbooks (24–26). Multisensory research is dominated by audiovisual research whereas audiotactile interactions, that is, how tones or noise bursts affect roughness perception (27), are scarce.

Magnetoencephalography has a huge advantage over fMRI especially in auditory and audiotactile domain since MEG acquisition is practically silent whereas fMRI involves concomitant high-intensity ambient noise associated with gradient coils and cryocooler. In addition, direct coupling to neuronal activity facilitates MS precision in MEG, and thus very detailed investigations related to neuronal processing involved.

We started to study the brain mechanisms underlying the largely unexplored audiotactile interactions in MEG in the 90's. Obviously, these experiments also required a novel MEG-compatible vibrotactile stimulation device.

It all started with an authentic audiotactile illusion discovered while testing an MEG-compatible microphone system. We coined the illusion as a parchment skin illusion (28) in which concomitant auditory feedback of the self-performed hand rubbing sound changes the perceived tactile sensation of the hands. The illusion is an excellent example of multisensory top–down processing in the brain. Later, the parchment skin illusion has been listed as one of the seven ways to fool your sense of touch freaky feelings (29) by New Scientist magazine. Charles Spence with his coauthors has exploited audiotactile illusions utilizing similar approaches in multisensory studies concerning, for example, roughness estimation (30) and crispness and staleness of potato chips (31) which earned them the Ig Nobel Prize in 2008.

**Figure 1:**
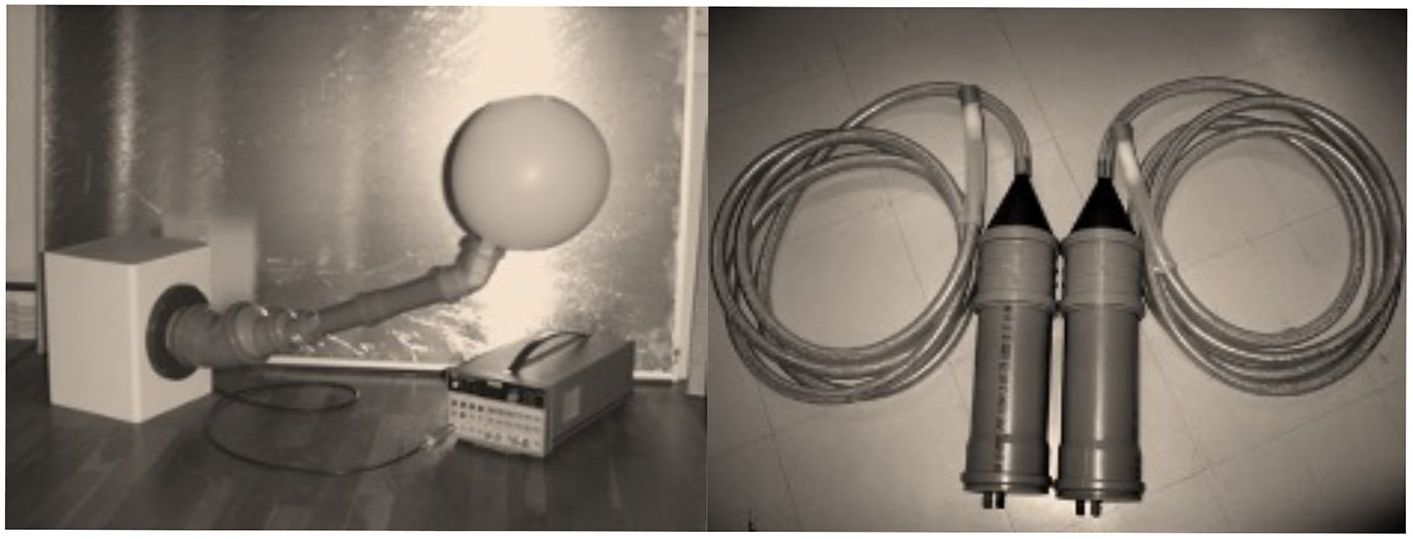
Left: The evolution of the house-built vibrotactile stimulator. The original vibrotactile stimulator was used to investigate a congenitally deaf adult (32). Right: The evolution version with a dedicated band-pass (100–500 Hz) filter was used later (33–35). Note that the original version has a balloon which vibrates by sound elicited by a standard loudspeaker whereas the later version uses a blind-ended silicone tube contributing to the reduced auditory contamination. White noise masking was typically used to reduce the auditory contamination.

I have learned audiotactile interactions in my childhood while playing with my two congenitally deaf cousins. Deaf persons use their tactile systems, that is, mechanoreceptors on the skin, for example, to efficiently control their voice and listen to music. With this background, we conducted a very unconventional experiment to demonstrate the activation of the auditory cortices in response to vibrotactile stimulation in a congenitally deaf adult. My new vibrotactile stimulator (see Figure 1), was crucial for the success of this study, which resulted in the first MEG publication showing a novel evidence on the plasticity in the auditory cortices in a congenitally deaf adult (32).

Investigations on audiotactile interactions with the vibrotactile stimulator have shed light on how hands help and activate auditory cortices in normal hearing subjects by means of MEG and fMRI recordings (33–35).

I consider that devices based on audiotactile interactions could be used efficiently in improving speech perception in noisy environments and hearing-disabled persons. In addition, such devices could be useful for rehabilitation purposes.

## Tactile Stimulation

The human tactile system provides us with an amazing spectrum of feedback, which enables us to perform tasks that require utterly precise motor control, such as playing musical instruments, and to sense minute vibrations. Touch even carries social and affective information (36) which is essential in our non-verbal communication. Unfortunately, fine-tuned tactile MEG-compatible stimulators are not readily available, largely preventing investigation of the tactile system with such ecologically-relevant stimuli.

**Figure 2:**
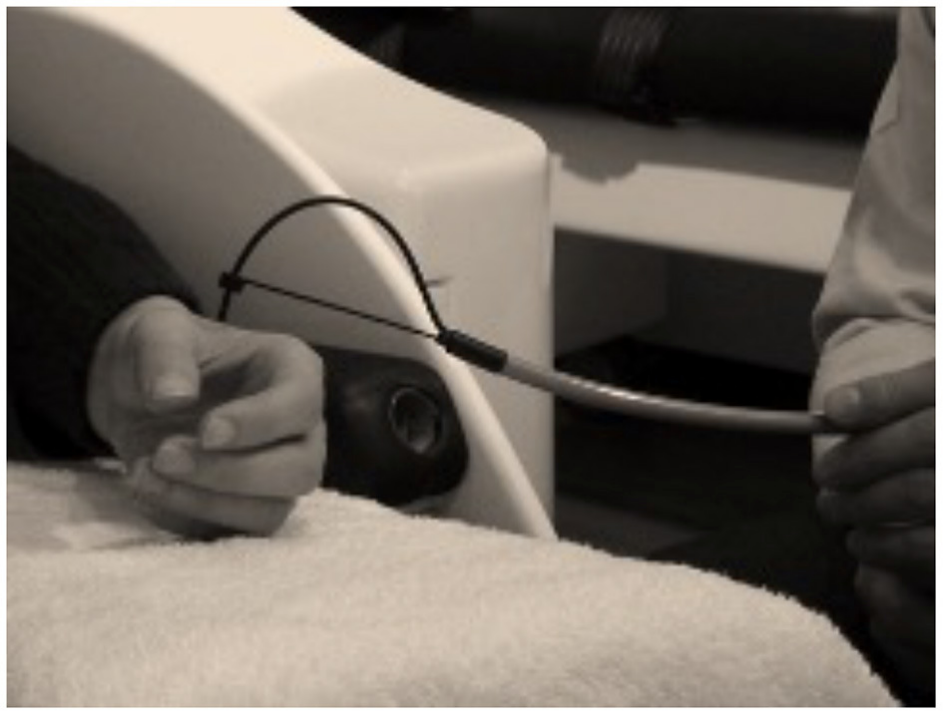
The original design for the tactile stimulator, a.k.a. woodpecker, used in tactile stimulation studiers (37, 39) is based on multifilament optical fiber (Schott Spectraflex; Schott AG, Mainz, Germany) and photosensor (Omron, Osaka, Japan). Note that the design of the handle limits the maximum force on the skin similarly as in an aesthesiometer based on von Frey filaments.

My original motivation was to find a way to get a precise trigger from the onset of the touch associated with von Frey monofilaments, which is used commonly for testing sensory thresholds of the human skin. It would open new pathways to study subthreshold tactile stimulation in MEG. Finally, I managed to discover a working solution comprising of a multifilament optic cable (Schott AG, Mainz, Germany) and an optosensor (Omron, Osaka, Japan) (see Figure 2). Multifilament optic cables consisting of hundreds of 50-μm fibers are used commonly for lightning in harsh environments. The multifilament cables are rather flexible and usable for infrared and visible light without any major attenuation. My approach is based on the idea that multifilament optic cable can be divided into two halves—one half for emitting the light from the optosensor and the other half to detect the reflection from the object. This innovation allowed us to generate a trigger from the skin contact at an accuracy of 1 mm in MEG recordings.

The first experiments with the novel brush stimulator, as we coined it at the time, were carried out at the National Rehabilitation Center (Tokorozawa, Japan). We used the brush stimulator to stimulate skin at the fingertip and lip and located the corresponding cortical sources (37). Later, the same approach has been used in several unique experiments shedding light on differences in pure observing and self vs. externally produced tactile stimulation with accurate and precise tactile stimulation in MEG (38, 39).

## Sensorimotor Mapping

As we know, motor cortices control actual movements, and peripheral feedback is used to fine-tune motor actions continuously. Such a closed-loop offers interesting options for monitoring efference and afference involved.

Magnetoencephalography has been used for functional sensorimotor mapping. Unfortunately, MEG recordings may be disturbed by large movements during the recordings, and thus, motor activities are typically limited to isometric muscle contractions or finger and foot movements. These issues can be mitigated using appropriate signal processing algorithms to compensate head movements, for example, signal space separation method (17). Such methods produce sufficient MEG signal quality to compensate low-frequency, smooth body movements but are limited to compensate for strong, brisk, and fast body movements.

Clinical practice guidelines list several protocols, for example, recording the premovement shift and corticomuscular coherence (CMC), for evaluating and locating motor cortices in MEG (1, 40). Typically, protocols require cooperation and results depend on the subject's performance level. In particular, disabled subjects may find these protocols very difficult to perform. On the other hand, motor mapping is clinically important in the preoperative evaluation of patients undergoing neurosurgery and could also be used during rehabilitation following a stroke or accident.

My motivation was to find an alternative solution for motor mapping using accelerometers to combine hand movements and MEG signals. The first accelerometers, for example, 40G Motorola accelerometers, that I tested in the late 90's were designed for the car industry and were far too magnetic and insensitive for the purpose. Ten years later, I stumbled upon an MEG-compatible 3D accelerometer ADXL330 (Analog Devices Inc., Norwood, MA) with analog output and 3G range—such accelerometers were used, for example, in Wii remote by Nintendo Co (Osaka, Japan). The component itself is non-magnetic although the operating current introduces some magnetic signals at a close distance, say within 50 cm from the MEG sensors.

At first, I envisioned three uses of the accelerometer in MEG: monitoring self-paced hand movements, monitoring the fundamental frequency of the voice, and using it as a response pad. Soon, we discovered that a similar approach had been already used to detect the onset of the motor movements (41). We set out to investigate possibilities for motor mapping using an accelerometer to record continuous self-paced movements at the Hôpital Erasme (Université Libre de Bruxelles, Brussels, Belgium).

**Figure 3:**
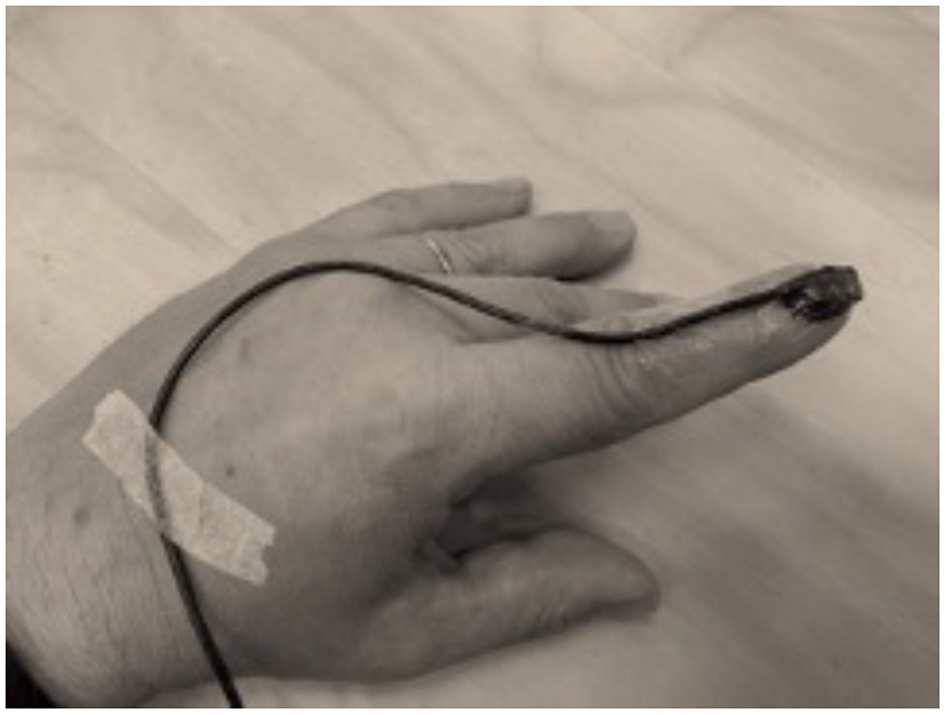
A typical setting in corticokinematic coherence studies (42–44) with a lightweight accelerometer (ADXL335; Analog Devices Inc, Norwood, MA) attached on the finger nail to pick up hand movements. Note that flexible cable allows natural hand movements.

We conducted measurements in MEG with an accelerometer attached to the finger (see Figure 3), whereas the subject was mimicking Parkinsonian tremor for three min. We could easily see a systematic coherence between the accelerometer and MEG signals. This discovery heralded the use of a new method to locate and monitor the activity at the primary somatomotor cortices during active and passive movements, and we coined the approach as corticokinematic coherence (CKC) in which coherence is calculated between movement kinematics monitored with an accelerometer and MEG signals (42).

Corticokinematic coherence studies published have shed light to address, for example, self-paced and externally paced movements (43), kinematics of the movements at various movement rates, and comparisons between hand-action-related acceleration, force, pressure, and electromyogram as a reference for CKC (44). CKC seems to reflect mainly movement-related proprioceptive afference (45), and thus, it is a very attracting tool to study proprioceptive systems in healthy and disabled subjects. In addition, CMC and CKC methods seem to complement each other (46). CKC studies have also provided a starting point to a possible bedside testing protocol to assess sensorimotor integration in newborns (47, 48).

Magnetoencephalography -compatible accelerometers have also been used successfully to study speech perception in humans to address coupling to the speech real-life situations (49–51). In addition, such accelerometers can be used to pick up utterances in a language testing paradigm in transcranial magnetic stimulation studies (52).

**Figure 4:**
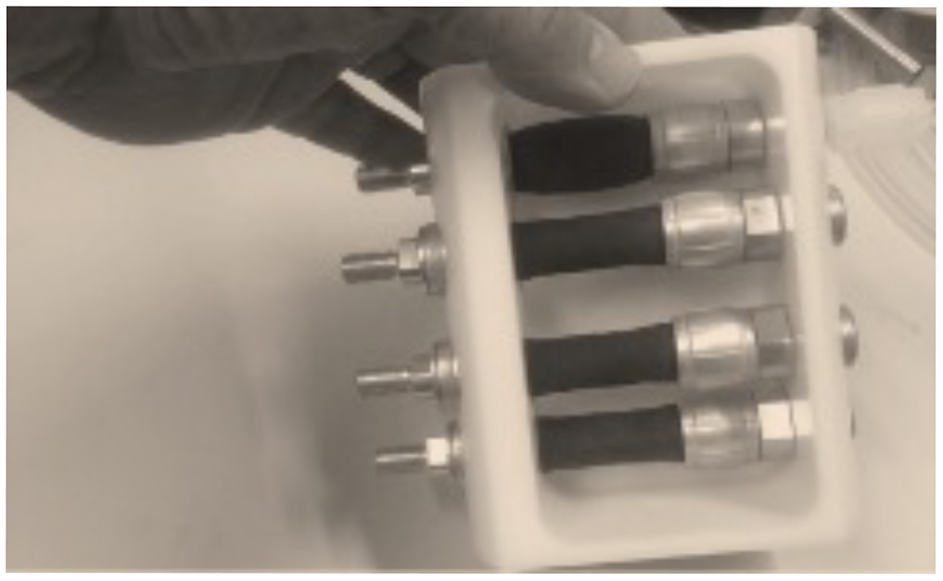
A 4-channel movement actuator system based on PAMs. Similar PAMs were used in studies using computer-controlled PAM stimulator. Note that the uppermost artificial muscle is contracting due to compressed air pulse applied on the muscle and the muscle is relaxed when it is depressurized. The maximum movement range of the muscle in the figure is about 10 mm, that is, 20% of the original length.

Since both self-paced and externally paced movements activate the same network in the brain, a computer-controlled stimulator for delivering precise and accurate finger movements comes very attractive. I discovered pneumatic artificial muscles (PAMs), originally invented in the 50's. These actuators are like badly designed pneumatic tubes expanding and shortening when pressurized (see Figure 4). Aramid fibers in the tube will push the muscle to its original length when the pressure is released. Such an actuator can be easily controlled by pneumatic relays outside the MSR.

**Figure 5:**
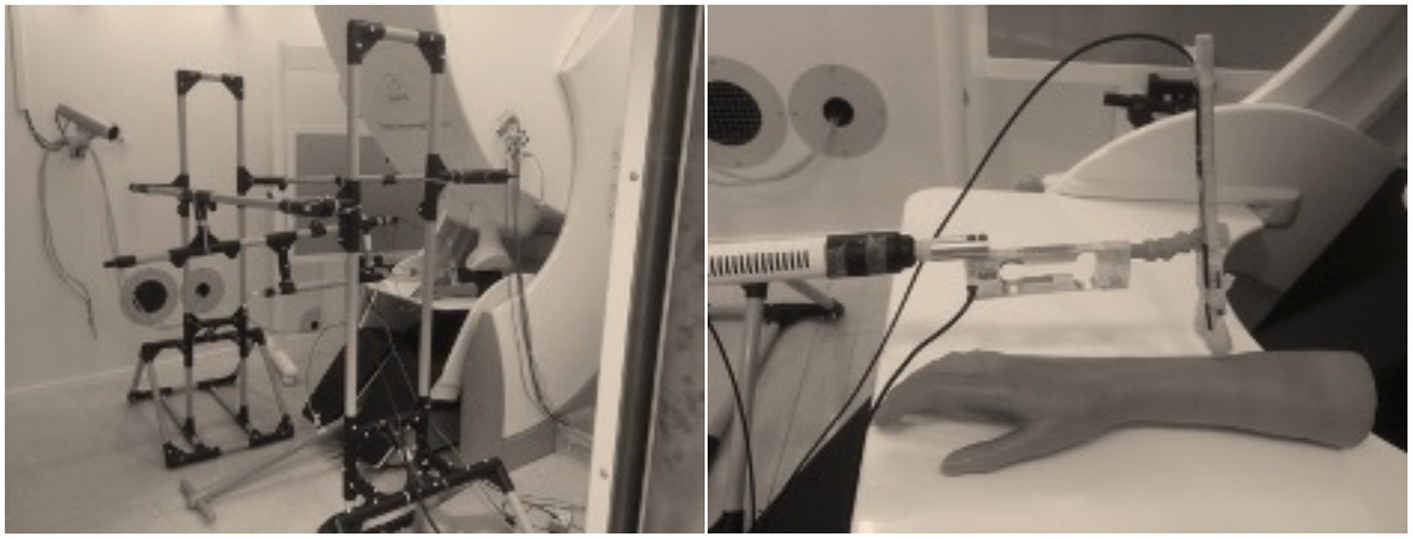
Left: The brush robot at NatMEG (Karolinska Institutet, Stockholm, Sweden) used to study gentle touch, that is, slowly conducting tactile (CT) fibers (57). Right: The brush robot uses similar PAMs as the PAM stimulator as the device in the Figure 4. Note that the computer-controlled device contains two PAMs for lifting and moving the brush, load cell to measure the force applied on the skin, accelerometer to monitor the movement, and two multifilament optic fibers to pick up the velocity of the brush movement and skin contact of the brush.

Pneumatic artificial muscle-based stimulators have been used in MEG, for example, in healthy subjects (53), Parkinson's patients (54), and Friedreich ataxia patients (55, 56) to explain proprioceptive afference and its impairment. Another PAM-based device (see Figure 5), has been used in investigations on slowly conducting tactile, that is, CT fibers contributing to gentle touch in MEG (57). In addition, PAM-based devices have been successfully used in fMRI studies (58–60).

## Intermittent Photic Stimulation

Clinical practice guidelines in MEG (https://www.acmegs.org/clinical-resources/practice-guidelines) define widely accepted clinical practices and provide an excellent view to the present state in clinical MEG. Comparison of the clinical practice guidelines in EEG (https://www.acns.org/practice/guidelines) indicates that MEG is still limited in use since clinical EEG has several applications that MEG misses. For example, intermittent photic stimulation (IPS) test is a vital part of clinical EEG with benefits whereas it is not mentioned in clinical MEG since commercial MEG-compatible IPS devices are not available.

Intermittent photic stimulation test is used in clinical EEG to study the cortical excitability during eyes open and eyes closed conditions (61). In patients with photosensitive epilepsy, IPS may cause epileptiform activity and even epileptic seizures (62).

Intermittent photic stimulation stimulators have progressed from the early xenon-based stimulator to modern LED-based devices (63). However, both types of IPS devices are not MEG compatible.

**Figure 6:**
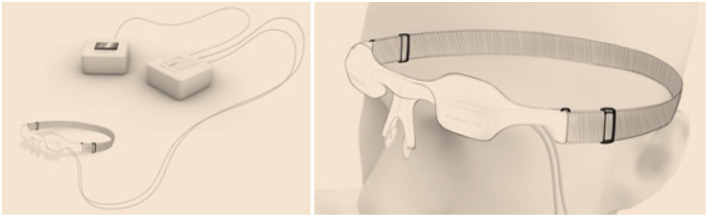
The design sketch of the Euphotic intermittent photic stimulation device (patents pending) using a unique diffuse light concept both in eyes-open and eyes-closed conditions. Note that the Euphotic IPS device allows unique option for simultaneous visual stimulation in eyes-open condition.

The idea to introduce novel IPS stimulator was triggered by the missing definition IPS in MEG in clinical practice guidelines. In this case, I filed an invention disclosure at Aalto University, and we have filed US and European patent applications to protect the ideas for potential commercial use. The euphotic team at Aalto University (Espoo, Finland) is developing the novel diffuse light concept in IPS further. With the novel Euphotic IPS device (see Figure 6), it is feasible to stimulate one or two eyes at the same time and use diffuse light both in eyes-open and eyes-closed conditions. In addition, it is a portable system and does not require eye fixation or focusing on the IPS device. The Euphotic IPS device is fully MEG compatible.

At present, the Euphotic project has established a preliminary business plan to take the authentic idea further and faster than in my previous innovations. The Euphotic project aims to collect patient and normative database and analysis tools to help to introduce IPS in clinical MEG.

Feasibility studies with Euphotic IPS device have ethical approvals at Cognitive Neuroimaging Centre (Nanyang Technological University, Singapore) and Aalto NeuroImaging (Aalto University, Espoo, Finland). Unfortunately, the COVID-19 outbreak has caused severe delays in MEG measurements in healthy subjects both in Singapore and Finland.

## Conclusions

As I have shown above, investigating sensory systems with natural, ecologically-relevant stimuli is feasible in MEG. First, Pacinian corpuscles can be selectively stimulated in MEG, and such stimuli can be utilized to study audiotactile interactions in congenitally deaf and normal hearing subjects. These novel findings help us to understand plasticity in the brain and how tactile sense affects the auditory sense and vice versa. Second, glabrous skin and lips can be stimulated with natural, ecologically relevant stimuli with MS precision in MEG. Such stimuli could be utilized in presurgical mapping and to monitor the recovery of peripheral nerve damages since given axons and associated sensory perceptions recover gradually. Third, active and passive movements can be investigated by utilizing accelerometers in MEG. The CKC method developed has proven to be very useful in investigating sensorimotor processing in healthy and diseased. Specifically, passive movements produced by MEG-compatible actuators, that are, PAMs, offer novel possibilities for presurgical mapping and designing novel experiments in MEG and fMRI. For example, such stimulators can be utilized to investigate gentle touch, that is, CT afferents, in MEG with ms precision. Fourth, the novel approach to use diffuse flickering light introduces MEG-compatible IPS device and opens novel ways to analyze cortical responses in epilepsy and healthy subjects.

These results mentioned above may open new avenues in research and translational clinical applications. It is important to notice that these steps from the bench to bedside involve multidisciplinary collaborators, time, effort, and reasonable funding.

Unfortunately, the gizmos that I have described here have not been FDA nor CE cleared, and thus, they are for investigational use only. We have plans to commercialize the Euphotic IPS device and we have a plan to collect evidence for the approvals in the forthcoming multicenter MEG study. We also have a plan to apply for FDA approval for the Euphotic IPS as a Class II medical device.

Both basic research and IPR-based solutions seem to work although the documentation load in the latter is elevated. On the other hand, IPR-based solution opens new paths for potential commercialization in the future once the evidence is available. The potential market could be easily expanded by designing stimulators and monitoring devices to be compatible with MEG and fMRI.

One of the major limiting factors for realizing novel ideas and stimulators is funding for basic research projects including preparing a prototype, creating preliminary documentation, initial recordings, and evidence to show that the idea works in reality. Universities would be optimal research sites for these steps whereas commercialization projects typically require a new company or contributions from well-established companies. Special research-to-business funding is available in several countries to facilitate the processes although such secured funding is limited and heavily competed. It also requires a realistic market estimate, strong business plan, global partners, and clear focus area. On the other hand, the valuation task can be very demanding since clinical practice guidelines do not support, for example, IPS in clinical MEG.

As shown above, I have created several gizmos for basic research and some of them are taking the first steps from the bench to the bedside. It has been fun and I have really enjoyed the work and collaboration with my global network.

## Author Contributions

The author confirms being the sole contributor of this work and has approved it for publication.

## Funding

Euphotic project at the Aalto University has financial support from the Aalto Brain Centre (https://www.aalto.fi/en/school-of-science/aalto-brain-centre).

## Conflict of Interest

VJ is heading the Euphotic team at Aalto University (Espoo, Finland). The euphotic team aims at commercializing the Euphotic IPS device. The Euphotic team is SPARK Finland SPARKee (https://sparkfinland.fi; Batch 2020). The immaterial rights of the Euphotic project belong to Aalto University.

## Publisher's Note

All claims expressed in this article are solely those of the authors and do not necessarily represent those of their affiliated organizations, or those of the publisher, the editors and the reviewers. Any product that may be evaluated in this article, or claim that may be made by its manufacturer, is not guaranteed or endorsed by the publisher.

## References

[1] Burgess RC Funke ME Bowyer SM Lewine JD Kirsch HE Bagic AI. American Clinical Magnetoencephalography Society Clinical Practice Guideline 2: presurgical functional brain mapping using magnetic evoked fields. J Clin Neurophysiol. (2011) 28:355–61. 10.1097/WNP.0b013e3182272ffe 21811122 PMC3366725

[2] Bagic AI. Disparities in clinical magnetoencephalography practice in the United States: a survey-based appraisal. J Clin Neurophysiol. (2011) 28:341–7. Available online at: https://journals.lww.com/clinicalneurophys/Abstract/2011/08000/Disparities_in_Clinical_Magnetoencephalography.3.aspx 21928507

[3] De Tiège X Lundqvist D Beniczky S Seri S Paetau R. Current clinical magnetoencephalography practice across Europe: Are we closer to use MEG as an established clinical tool? Seizure. (2017) 50:53–9. 10.1016/j.seizure.2017.06.002 28623727

[4] Baud MO Perneger T Racz A Pensel MC Elger C Rydenhag B. European trends in epilepsy surgery. Neurology. (2018) 91:e96–106. 10.1212/WNL.0000000000005776 29898967

[5] Mouthaan BE Huiskamp GM Leijten FS Braun KP. Response to: Guidelines for the clinical use in epilepsy surgery evaluation of magnetoencephalography and electroencephalography for source localization. Epilepsia. (2016) 57:1942. 10.1111/epi.13581 27869998

[6] Shiraishi H Ozaki I Igushi Y Ishii R Kamada K Kameyama S. Questionnaire survey of current status and problems in clinical applications of magnetoencephalography (MEG) in Japan. Jpn J Clin Neurophysiol. (2012) 40:119–30. 10.11422/jscn.40.119

[7] Bagic AI Burgess RC. Clinical magnetoencephalography practice in the United States ten years later: a survey-based reappraisal. J Clin Neurophysiol. (2020) 37:592–8. 10.1097/WNP.0000000000000693 33165232

[8] de Lange P Boto E Holmes N Hill RM Bowtell R Wens V. Measuring the cortical tracking of speech with optically-pumped magnetometers. Neuroimage. (2021) 233:117969. 10.1016/j.neuroimage.2021.117969 33744453

[9] Shah VK Wakai RT. A compact, high performance atomic magnetometer for biomedical applications. Phys Med Biol. (2013) 58:8153–61. 10.1088/0031-9155/58/22/8153 24200837 PMC3971838

[10] Boto E Bowtell R Kruger P Fromhold TM Morris PG Meyer SS. On the potential of a new generation of magnetometers for MEG: A Beamformer simulation study. PLoS One. (2016) 11:e0157655. 10.1371/journal.pone.0157655 27564416 PMC5001648

[11] Boto E Meyer SS Shah V Alem O Knappe S Kruger P. A new generation of magnetoencephalography: Room temperature measurements using optically-pumped magnetometers. Neuroimage. (2017) 149:404–14. 10.1016/j.neuroimage.2017.01.034 28131890 PMC5562927

[12] Tierney TM Holmes N Mellor S Lopez JD Roberts G Hill RM. Optically pumped magnetometers: From quantum origins to multi-channel magnetoencephalography. Neuroimage. (2019) 199:598–608. 10.1016/j.neuroimage.2019.05.063 31141737 PMC6988110

[13] Kominis IK Kornack TW Allred JC Romalis MV. A subfemtotesla multichannel atomic magnetometer. Nature. (2003) 422:596–9. 10.1038/nature01484 12686995

[14] Hari R Puce A. MEG-EEG Primer. New York, NY: Oxford University Press (2017).

[15] Hämäläinen M Hari R Ilmoniemi RJ Lounasmaa OV. Magnetoencephalography-theory, instrumentation, and applications to noninvasive studies of the working human brain. Rev Modern Phys. (1993) 65:413–97. 10.1103/RevModPhys.65.413

[16] Burgess RC. Recognizing and correcting MEG artifacts. J Clin Neurophysiol. (2020) 37:508–17. 10.1097/WNP.0000000000000699 33165224

[17] Taulu S Kajola M Simola J. Suppression of interference and artifacts by the signal space separation method. Brain Topogr. (2004) 16:269–75. 10.1023/B:BRAT.0000032864.93890.f9 15379226

[18] Taulu S Hari R. Removal of magnetoencephalographic artifacts with temporal signal-space separation: demonstration with single-trial auditory-evoked responses. Hum Brain Mapp. (2009) 30:1524–34. 10.1002/hbm.20627 18661502 PMC6871056

[19] Bourguignon M Whitmarsh S Piitulainen H Hari R Jousmäki V Lundqvist D. Reliable recording and analysis of MEG-based corticokinematic coherence in the presence of strong magnetic artifacts. Clin Neurophysiol. (2016) 127:1460–9. 10.1016/j.clinph.2015.07.030 26337839

[20] Airaksinen K Makela JP Taulu S Ahonen A Nurminen J Schnitzler A. Effects of DBS on auditory and somatosensory processing in Parkinson's disease. Hum Brain Mapp. (2011) 32:1091–9. 10.1002/hbm.21096 20645306 PMC6870287

[21] Abbasi O Hirschmann J Schmitz G Schnitzler A Butz M. Rejecting deep brain stimulation artefacts from MEG data using ICA and mutual information. J Neurosci Methods. (2016) 268:131–41. 10.1016/j.jneumeth.2016.04.010 27090949

[22] Kandemir AL Litvak V Florin E. The comparative performance of DBS artefact rejection methods for MEG recordings. Neuroimage. (2020) 219:117057. 10.1016/j.neuroimage.2020.117057 32540355 PMC7443703

[23] Kakisaka Y Mosher JC Wang ZI Jin K Dubarry AS Alexopoulos AV. Utility of temporally-extended signal space separation algorithm for magnetic noise from vagal nerve stimulators. Clin Neurophysiol. (2013) 124:1277–82. 10.1016/j.clinph.2012.03.082 22727713 PMC3980452

[24] Calvert GA Spence C Stein BE. The Handbook of Multisensory Processes. Cambridge, MA: A Bradford Book (2004).

[25] Stein BE Meredith MA. The Merging of the Senses. Cambridge, MA: A Bradford Book;. (2004).

[26] Stein BE. The New Handbook of Multisensory Processing. Cambridge, MA: A Bradford Book (2012).

[27] Schiller P. Die Rauhigkeit als intermodale Erscheinung. Z Psychol Bd. (1932) 127:265–89.

[28] Jousmäki V Hari R. Parchment-skin illusion: sound-biased touch. Curr Biol. (1998) 8:R190. 10.1016/S0960-9822(98)70120-4 9512426

[29] Lawton G. That freaky feeling. New Sci. (2009) 14:32–27. 10.1016/S0262-4079(09)63252-829940202

[30] Guest S Catmur C Lloyd D Spence C. Audiotactile interactions in roughness perception. Exp Brain Res. (2002) 146:161–71. 10.1007/s00221-002-1164-z 12195518

[31] Zampini M Spence C. The role of auditory cues in modulating the perceived crispness and staleness of potato chips. J Sensory Stud. (2004) 19:347–63. 10.1111/j.1745-459x.2004.080403.x

[32] Levänen S Jousmäki V Hari R. Vibration-induced auditory-cortex activation in a congenitally deaf adult. Curr Biol. (1998) 8:869–72. 10.1016/S0960-9822(07)00348-X 9705933

[33] Schürmann M Caetano G Jousmäki V Hari R. Hands help hearing: facilitatory audiotactile interaction at low sound-intensity levels. J Acoust Soc Am. (2004) 115:830–2. 10.1121/1.1639909 15000194

[34] Caetano G Jousmäki V. Evidence of vibrotactile input to human auditory cortex. Neuroimage. (2006) 29:15–28. 10.1016/j.neuroimage.2005.07.023 16168673

[35] Schürmann M Caetano G Hlushchuk Y Jousmäki V Hari R. Touch activates human auditory cortex. Neuroimage. (2006) 30:1325–31. 10.1016/j.neuroimage.2005.11.020 16488157

[36] Olausson H Wessberg J Morrison I McGlone F Vallbo A. The neurophysiology of unmyelinated tactile afferents. Neurosci Biobehav Rev. (2010) 34:185–91. 10.1016/j.neubiorev.2008.09.011 18952123

[37] Jousmäki V Nishitani N Hari R. A brush stimulator for functional brain imaging. Clin Neurophysiol. (2007) 118:2620–4. 10.1016/j.clinph.2007.08.024 17950032

[38] Pihko E Nangini C Jousmäki V Hari R. Observing touch activates human primary somatosensory cortex. Eur J Neurosci. (2010) 31:1836–43. 10.1111/j.1460-9568.2010.07192.x 20584188

[39] Hesse MD Nishitani N Fink GR Jousmäki V Hari R. Attenuation of somatosensory responses to self-produced tactile stimulation. Cereb Cortex. (2010) 20:425–32. 10.1093/cercor/bhp110 19505992

[40] De Tiège X Bourguignon M Piitulainen H Jousmäki V. Sensorimotor mapping with MEG: An update on the current state of clinical research and practice with considerations for clinical practice guidelines. J Clin Neurophysiol. (2020) 37:564–73. 10.1097/WNP.0000000000000481 33165229

[41] Bowyer SM Mason K Yaeger BJW Moran JE Barkley GL Tepley N. Localization of motor cortex by MEG using a tremorometer. Int Congress Series. (2007) 1300:321–4. 10.1016/j.ics.2007.02.001

[42] Bourguignon M De Tiège X Op de Beeck M Pirotte B Van Bogaert P Goldman S. Functional motor-cortex mapping using corticokinematic coherence. Neuroimage. (2011) 55:1475–9. 10.1016/j.neuroimage.2011.01.031 21256222

[43] Piitulainen H Bourguignon M De Tiège X Hari R Jousmäki V. Corticokinematic coherence during active and passive finger movements. Neuroscience. (2013) 238:361–70. 10.1016/j.neuroscience.2013.02.002 23402851

[44] Piitulainen H Bourguignon M De Tiège X Hari R Jousmäki V. Coherence between magnetoencephalography and hand-action-related acceleration, force, pressure, and electromyogram. Neuroimage. (2013) 72:83–90. 10.1016/j.neuroimage.2013.01.029 23357073

[45] Bourguignon M Piitulainen H De Tiege X Jousmäki V Hari R. Corticokinematic coherence mainly reflects movement-induced proprioceptive feedback. Neuroimage. (2015) 106:382–90. 10.1016/j.neuroimage.2014.11.026 25463469 PMC4295920

[46] Bourguignon M Jousmäki V Dalal SS Jerbi K De Tiège X. Coupling between human brain activity and body movements: Insights from non-invasive electromagnetic recordings. Neuroimage. (2019) 203:116177. 10.1016/j.neuroimage.2019.116177 31513941

[47] Vanhatalo S Jousmäki V Andersson S Metsäranta M. An easy and practical method for routine, bedside testing of somatosensory systems in extremely low birth weight infants. Pediatr Res. (2009) 66:710–3. 10.1203/PDR.0b013e3181be9d66 19730159

[48] Smeds E Vanhatalo S Piitulainen H Bourguignon M Jousmäki V Hari R. Corticokinematic coherence as a new marker for somatosensory afference in newborns. Clin Neurophysiol. (2017) 128:647–55. 10.1016/j.clinph.2017.01.006 28237690

[49] Vander Ghinst M Bourguignon M Op de Beeck M Wens V Marty B Hassid S. Left superior temporal gyrus is coupled to attended speech in a cocktail-party auditory scene. J Neurosci. (2016) 36:1596–606. 10.1523/JNEUROSCI.1730-15.2016 26843641 PMC6601992

[50] Vander Ghinst M Bourguignon M Niesen M Wens V Hassid S Choufani G. Cortical tracking of speech-in-noise develops from childhood to adulthood. J Neurosci. (2019) 39:2938–50. 10.1523/JNEUROSCI.1732-18.2019 30745419 PMC6462442

[51] Bourguignon M De Tiège X de Beeck MO Ligot N Paquier P Van Bogaert P. The pace of prosodic phrasing couples the listener's cortex to the reader's voice. Hum Brain Mapp. (2013) 34:314–26. 10.1002/hbm.21442 22392861 PMC6869855

[52] Vitikainen AM Mäkelä E Lioumis P Jousmäki V Mäkelä JP. Accelerometer-based automatic voice onset detection in speech mapping with navigated repetitive transcranial magnetic stimulation. J Neurosci Methods. (2015) 253:70–7. 10.1016/j.jneumeth.2015.05.015 26026582

[53] Piitulainen H Bourguignon M Hari R Jousmäki V. MEG-compatible pneumatic stimulator to elicit passive finger and toe movements. Neuroimage. (2015) 112:310–7. 10.1016/j.neuroimage.2015.03.006 25770989

[54] Vinding MC Tsitsi P Piitulainen H Waldthaler J Jousmäki V Ingvar M. Attenuated beta rebound to proprioceptive afferent feedback in Parkinson's disease. Sci Rep. (2019) 9:2604. 10.1038/s41598-019-39204-3 30796340 PMC6385616

[55] Naeije G Bourguignon M Wens V Marty B Goldman S Hari R. Electrophysiological evidence for limited progression of the proprioceptive impairment in Friedreich ataxia. Clin Neurophysiol. (2020) 131:574–6. 10.1016/j.clinph.2019.10.021 31839397

[56] Marty B Naeije G Bourguignon M Wens V Jousmäki V Lynch DR. Evidence for genetically determined degeneration of proprioceptive tracts in Friedreich ataxia. Neurology. (2019) 93:e116–24. 10.1212/WNL.0000000000007750 31197032

[57] Eriksson Hagberg E Ackerley R Lundqvist D Schneiderman J Jousmäki V Wessberg J. Spatio-temporal profile of brain activity during gentle touch investigated with magnetoencephalography. Neuroimage. (2019) 201:116024. 10.1016/j.neuroimage.2019.116024 31323258

[58] Lolli V Rovai A Trotta N Bourguignon M Goldman S Sadeghi N. MRI-compatible pneumatic stimulator for sensorimotor mapping. J Neurosci Methods. (2019) 313:29–36. 10.1016/j.jneumeth.2018.12.014 30578869

[59] Lolli V Rovai A Trotta N Goldman S Sadeghi N Lefranc F. Pneumatic artificial muscle-based stimulator for passive functional magnetic resonance imaging sensorimotor mapping in patients with brain tumours. J Neurosci Methods. (2021) 359:109227. 10.1016/j.jneumeth.2021.109227 34052287

[60] Nurmi T Henriksson L Piitulainen H. Optimization of proprioceptive stimulation frequency and movement range for fMRI. Front Hum Neurosci. (2018) 12:477. 10.3389/fnhum.2018.00477 30559657 PMC6286983

[61] Tatum WO Rubboli G Kaplan PW Mirsatari SM Radhakrishnan K Gloss D. Clinical utility of EEG in diagnosing and monitoring epilepsy in adults. Clin Neurophysiol. (2018) 129:1056–82. 10.1016/j.clinph.2018.01.019 29483017

[62] Martins da Silva A Leal B. Photosensitivity and epilepsy: Current concepts and perspectives-A narrative review. Seizure. (2017) 50:209–18. 10.1016/j.seizure.2017.04.001 28532712

[63] Kasteleijn-Nolst Trenite D Carr B Checa-Ros A Seri S. Light-emitting-diode and Grass PS 33 xenon lamp photic stimulators are equivalent in the assessment of photosensitivity: Clinical and research implications. Epilepsy Res. (2020) 165:106377. 10.1016/j.eplepsyres.2020.106377 32505867

